# Innovative Process for Dried Caper (*Capparis spinosa* L.) Powder Production

**DOI:** 10.3390/foods11233765

**Published:** 2022-11-23

**Authors:** Fabrizio Cincotta, Maria Merlino, Antonella Verzera, Enrico Gugliandolo, Concetta Condurso

**Affiliations:** Department of Veterinary Sciences, University of Messina, Polo Universitario dell’Annunziata, Viale G. Palatucci, 98168 Messina, Italy

**Keywords:** *Capparis Spinosa* L. powder, production processes, sensory features, aroma compounds, consumer acceptability, antioxidant capacity

## Abstract

This research aimed to develop a new time, energy, and cost-saving production process for obtaining dried powder from *Capparis spinosa* floral buds. Four different trials, including dry salting with 40% NaCl (for 10 days and 40 days) and brine salting with 18% NaCl (at room temperature for 3 days and at 60 °C for 6 h), were carried out, and two different air-drying temperatures (40 and 50 °C) were used. The effects on chemical and sensory characteristics were investigated and compared with traditional undried caper samples. Spectroscopy and chromatographic techniques such as UV–VIS, GC-MS, and FTIR were used for chlorophylls, carotenoids, polyphenols, flavonoids, and volatile aroma compounds’ analyses. Moreover, a sensory descriptive analysis and acceptability were applied to individuate the product most appreciated by the consumers. Among the different trials, brine salting at 60 °C and drying at 50 °C constituted the fastest process that yielded an appreciated powder by consumers; the chemical analyses demonstrated that this process did not lead to the formation of extraneous aroma compounds that could influence the typical sensory properties of capers and maintained high levels of chlorophylls, carotenoids, and polyphenols. Altogether, the results could be of great significance to industrial production and potentiate positive impacts on the economy of production areas.

## 1. Introduction

In the last few years, the market of dried products has received considerable and increasing interest due to the fostered demand for healthy convenience foods with a longer shelf-life [1]. Dried fruits and vegetables are recognized as healthy for human consumption; the dehydration process, by lowering water activity, keeps fruits and vegetables healthy and safe, thereby prolonging their shelf-life [2]. Vegetable powders are widely used in many food sectors, mainly as functional ingredients for improving the nutritional value of a food product, as flavoring agents, or as colorants. The powder quality depends mainly on the drying method, but it is also dependent on the vegetable’s physical and chemical composition and characteristics [3]. Air-drying methods are the most widespread techniques for the dehydration of vegetables since they are simple and cost-effective [4]. Recent studies deal with saving time and energy in conventional and innovative methods to obtain dried powders from different vegetables such as ginger, turmeric, chili, black pepper cardamom, garlic, and onions [5,6]. However, a suitable drying technology must be selected in relation to the food matrix since it influences the powder’s quality, such as its rehydration capacity, loss of taste, aroma, color, and nutritional value [7]. For this reason, sensory analysis and antioxidant activity are usually used for the quality evaluation of dried vegetables and powders [8].

*Capparis spinosa* L. is a widespread plant throughout the Mediterranean area. Floral buds are its most used part. Before consumption, the buds are subjected to a dry-salting fermentation process that results in the development of volatile compounds that are responsible for its unique sensory features. The production process consists of a pre-treatment after harvesting, in which raw, fresh floral buds are mixed with marine salt (30–40% by weight) and shuffled for about 8 to 10 days. During this period, brine is formed, and fermentation occurs. Then, the brine is discarded, and the buds are treated with marine salt (20–25% by weight) for another 20 to 30 days to obtain the finished product, commonly referred to as capers [9]. The capers, usually packaged in plastic bags added with 25% marine salt by weight, must be sweetened by placing them in water to reduce their salt content before their consumption.

Recently, an increasing interest in dried caper powder has been observed by gastronomes, bartenders, and professional chefs that normally prepare it by themselves starting from the traditional capers; in fact, it is scarcely industrially produced and present on the market at a very high price. The reason is that the production of dried caper powder is not economically advantageous since a long fermentation process is added to the cost of the energy-consuming drying procedure [10]. Nevertheless, the fermentation process is a crucial step in capers’ aromatic and flavor development, and it cannot not be totally avoided; moreover, heat treatments can lead to the formation of compounds that interfere with capers’ typical sensory properties [11].

In the context of an increasing interest in dried vegetables, the industrial production of dried caper powder could be of considerable commercial interest for two main reasons: on the one hand, there is a reduction in mass weight and, therefore, the possibility to trade in larger volumes, and on the other hand, it is an easy-to-use product (without the need of any pretreatment) with a longer shelf-life when compared to traditional capers.

Regarding this matter, the aim of our research is to develop an innovative time, energy, and cost-saving production process to obtain a dried powder starting from the fresh *Capparis spinosa* L. buds with sensory features that are acceptable by consumers. To the best of our knowledge, no information is available in the literature on the chemical and sensory features of dried caper powder nor on its production process. To achieve this goal, different trials were carried out and their effects on the product’s sensory characteristics and consumer acceptability were investigated; UV–Vis Spectroscopy, Fourier Transformed Infrared Spectroscopy (FTIR), and Gas Chromatography-Mass Spectrometry (GC-MS) were applied for chemical characterization. Moreover, the product’s antioxidant capacity has been taken into consideration.

## 2. Materials and Methods

### 2.1. Material

A total of 4 kg of freshly picked floral buds were supplied by a local producer with a guarantee of authenticity. The samples were harvested in June 2021 from 20 different plants of *Capparis spinosa* L. cultivated in a wide geographic area situated in the west side of Lipari Island (Eolian archipelago, Sicily, Italy). Once collected, the samples were immediately transported to the laboratory and stored at room temperature in a cool, clean, well-ventilated, odorless, and dry place, away from direct sunlight and heat sources until the further processing phases.

### 2.2. Production Processes

The fresh buds were then divided into four aliquots, in which each one was subjected to a different production process. Exactly, two aliquots were subjected to brine salting (BrS) and the other two dry salting (DrS) before the following drying procedure was applied:

(1) BrS (Rt)—fresh buds were placed in brine (18% NaCl) at Room Temperature (~25 °C) for 3 days;
(2) BrS (60 °C)—fresh buds were placed in brine (18% NaCl) at 60 °C for 6 h;
(3) DrS (10d)—fresh buds were mixed with 40% of dry NaCl for 10 days;
(4) DrS (40d)—fresh buds mixed with 40% of dry NaCl for 10 days, and then the brine that had formed was discarded and 25% of dry NaCl was added and mixed for 30 days.

Brine concentration and brining time for the first two processes were set according to preliminary trials until the capers’ bitter taste was neutralized. 

Process consisting of applying n° 4 (DrS (40d)) is the traditional production process used to obtain capers (Cappero delle Isole Eolie” Protected Designation of Origin (PDO), whereas the process consisting of applying n° 3 (DrS (10d)) is the intermediate step of this process [12].

### 2.3. Drying Procedures

The samples BrS (Rt), BrS (60 °C), DrS (10d), and DrS (40d) were submitted to hot air drying in a tray dryer (Armfield Ltd., Model UOP8, Hampshire, England) at two different temperatures (40° and 50 °C) and at constant air velocity (1.5 m/s). Approximately 100 g of the samples, which was exactly weighted for each type, was distributed onto a metal tray (27 × 18.5 × 1.5 cm). Air temperature and velocity were continuously measured throughout the experiments. During the drying procedures, the samples were periodically removed from the dryer and weighed using an analytical balance (Sartorius mod. QUINTIX 65-1S, Göttingen Germany) with an accuracy of 0.0001 g. The drying procedures were concluded when a moisture loss of 96–98% against the raw samples had been reached. The drying procedures were repeated three times under the same conditions. Once the dried samples were obtained, they were milled in a home blender (La Moulinette, Moulinex, Écully, France, 2002) for 10 min. The milled powders were sieved using a set of sieves of different sizes coupled to vibrating support (model AS 200 basic, Retsch, Germany). For all the samples, most of the powders had a granulometry ranging between 35 and 60 mesh (0.5 and 0.25 mm). The powder fractions included in this range size were than analyzed in triplicate.

Samples not subjected to the drying procedure through the DrS (40d) process (undried capers) were also considered and analyzed. 

### 2.4. Moisture Content

The moisture content was determined by the oven-drying method, maintaining the samples at 110 ± 0.5 °C until a constant mass was achieved [13]. 

### 2.5. Chlorophylls and Carotenoids

Chlorophyll and carotenoid extraction were carried out according to the method reported by Tayiroğlu and İncedayı [14]. In detail, 1 g of powder was added to 9 mL of pure methanol and the mixture was kept in an ultrasonic water bath (Helma Transsonic T 460/H, Germany) for 2 hr. Then, the samples were centrifuged at 10,000 rpm for 10 min (Eppendorf Centrifuge 5810 R, Germany), and the extract absorbances were determined against pure methanol at 470 nm, 645 nm, and 662 nm wavelengths. The following formulas were used to determine the amount of chlorophyll a, chlorophyll b, total chlorophylls, and total carotenoids [15].

Chlorophyll a (Cla) = 11.75 × A662 − 2.35 × A645(1)

Chlorophyll b (Clb) = 18.61 × A645 − 3.96 × A662(2)

Total Chlorophyll = Cla + Clb(3)

Total Carotenoids = (1000 × A470 − 2.27 Cla − 81.4 Clb)/227(4)

### 2.6. Total Phenolics and Flavonoids

Total phenolic and flavonoid content has been determined following the method reported by Tayiroğlu and İncedayı [14]; specifically, 4.5 mL of 80% methanol was added to 2 g of sample and shaken for 2 h at 20 °C in a shaking water bath (Thermo Scientific™ TSSWB15 Precision SWB 15 model, Waltha, MA, USA), and then centrifuged at 10,000 rpm for 10 min (Eppendorf Centrifuge 5810 R, Hamburg, Germany). The supernatant was transferred to a different tube, and the residue was mixed with 4.5 mL of 80% methanol again and the same extraction steps were performed. The supernatants were combined and cold-stored until analysis.

The total phenolic content of the extract was determined using Folin–Ciocalteu reagent; a total of 0.25 mL of appropriately diluted sample was mixed with 1.25 mL of Folin–Ciocalteu reagent (1:10), and after 5 min, 1 mL of Na_2_CO_3_ (7.5%) was added. Then, the mixtures were vortexed and incubated in the dark for 60 min at room temperature. The absorbance at 765 nm was then measured against the prepared blank. The results were expressed in terms of mg Gallic Acid Equivalent (GAE)/g dry matter calculated by the regression equation of the calibration curve, which was obtained by analyzing different concentrations of gallic acid standard solutions in the range of 0.005–0.5 mg/mL.

Total flavonoids were analyzed using the colorimetric assay reported by Ordoñez et al. [16]; 1 mL of 2% AlCl_3_-methanol solution was added to 1 mL of diluted sample and the mixture was vortexed. After the reaction, the mixture was kept in the dark for 1 h and then the absorbance was determined against the control sample at 420 nm. The regression equation of the calibration curve, obtained by performing the same procedures with different concentrations of the standard quercetin solution (1–8 μg/mL), was used to calculate the total flavonoid concentration as mg Quercetin Equivalent (QE)/g dry matter.

### 2.7. Antioxidant Capacity

The Antioxidant Capacity (AC) of the methanolic extracts obtained in Section 2.6 was measured by a 2,2-diphenyl-1-picrylhydrazyl radical (DPPH) assay via a commercially available assay kit (Bockit, KF-01-007) according to the manufacturer’s protocol. Briefly, 50 μL of sample or trolox (positive control) were appropriately diluted in dimethyl sulfoxide (DMSO) and mixed with 1450 μL of DPPH radical solution (0.06 mM) provided by the kit. After 30 min of incubation at room temperature in the dark, the total Antioxidant Capacity (AC) was determined by measuring absorbance at 517 nm through a spectrophotometer (Multiskan™ FC Microplate Photometer, Thermo Scientific™) against pure methanol and calculating the corresponding percentage of the inhibition of the radical DPPH, as reported in the kit’s instructions, by using the following equation:

AC (%) = (AbsControl − AbsSample)/AbsControl × 100(5)

AbsControl: absorbance of sample-free DPPH solution; AbsSample: absorbance of DPPH solution containing the sample [17].

### 2.8. FTIR Analysis

FTIR spectroscopy analysis was carried out on the powder and undried caper samples to determine the nature and structure of different functional groups of their chemical compounds [18]. The infrared spectra were obtained from a Shimadzu IRAffinity-1S FTIR spectrophotometer (Shimadzu Italia S.r.l., Milan, Italy) equipped with a sealed and desiccated interferometer, a Deuterated Triglycine Sulphate Doped with L-Alanine (DLATGS) detector, and a Specac Quest Attenuated Total Reflectance (ATR) accessory (Specac Ltd., London, England). The acquisitions were made by performing 45 scans between 4000 and 350 cm^−1^, with a resolution of 4 cm^−1^. After scanning, the organic functional groups were identified through the location of the different bands on the FTIR spectrum and compared with those reported in the literature. 

### 2.9. Volatile Analysis Aroma Compounds Analysis

Volatile aroma compounds were analyzed by Head Space-Solid Phase Micro Extraction-Gas Chromatography-Mass Spectrometry (HS-SPME-GC-MS) following the method reported by Condurso et al. [9] with some modifications. A total of 0.1 g of each powder sample was placed into a 7 mL glass vial, supplemented with 3.5 mL of saturated NaCl solution, and equilibrated for 30 min at 35 °C. A triphasic DVB/CARB/PDMS fiber was then exposed to the headspace for 40 min for the extraction of volatile compounds and successively transferred to the injection port of the GC at 260 °C and maintained for 3 min. A Shimadzu GC 2010 Plus gas chromatographer coupled with a TQMS 8040 triple quadrupole mass spectrometer (Shimadzu, Milan, Italy) equipped with a CP-Wax-52-CB capillary column (60 m and 0.25 mm i.d.; coating thickness—0.25 μm) was used for the GC-MS analyses by using the following conditions: injector temperature, 260 °C; injection mode, splitless; oven temperature, 45 °C, which was held for 5 min, then increased to 80 °C at a rate of 10 °C/min, and to 240 °C at 2 °C/min; carrier gas, helium at a constant flow of 1 mL/min; transfer line temperature, 250 °C; acquisition range, 40–400 m/z; scan speed, 1250 amu/s.

The identification of the compounds was executed by mass spectral data, NIST’20 (NIST/EPA/NIH Mass Spectra Library, Wiley, Hoboken, NJ, USA) FFNSC 3.0 database, Linear Retention Indices (LRI), literature data, and the injection of the available standards. The LRI was calculated according to Van den Dool and Kratz’s equation. 

### 2.10. Sensory Analysis

Qualitative Descriptive Analysis (QDA) was carried out to evaluate the sensory characteristics of the dried powder and undried caper samples using a trained sensory panel consisting of 8 assessors (4 females and 4 males, aged between 24 and 50 years) recruited among students and workers of the Department of Veterinary Science of the University of Messina. The panel was trained according to ISO 8586:2012 [19]; a common vocabulary was developed in preliminary sessions for the explanation of the sensory descriptors and to familiarize the assessors with scales and procedures. Each descriptor was extensively described and explained to avoid any doubt about their specific meanings. Based on the frequency of citation, 21 descriptors were selected. The judges evaluated the intensity of each descriptor by assigning a score between 1 (absence of the sensation) and 9 (extremely intense). Each judge evaluated the caper samples in three sessions. All evaluations were carried out from 10.00 to 12.00 A.M. in individual booths illuminated by white light. The order of presentation was randomized among judges and sessions. Water and unsalted crackers were provided to judges between samples. All data were acquired by a direct computerized registration system (FIZZ Byosistemes. ver. 2.00 M, Couternon, France). 

### 2.11. Consumer Acceptability Test

Consumer acceptability was assessed randomly by consumers (*n* = 80; 37 males and 43 females; 24–60 years) who were selected among the students and personnel of the University of Messina. Before the assessment, they were invited to fill out a questionnaire to gauge their consumption frequency and appreciation of capers. Participation in the consumer survey was voluntary. Consumers evaluated the samples based on 4 attributes, namely, appearance, odor, taste, and overall acceptability. The scores were recorded based on the hedonic scale method, where 9 = like extremely and 1 = dislike extremely. After each trial, water was served to avoid inaccuracy due to the high similarities of the samples.

### 2.12. Statistical Analysis

Data were analyzed using XLStat software, version 2019.1.2 (Addinsoft, Damremont, Paris, France). Two-way Analysis of Variance (ANOVA) and Duncan’s multiple range test at a confidence level of 95% were applied to chemical data to determine significant differences among samples. Principal Component Analysis (PCA) was performed on aroma volatiles data.

## 3. Results and Discussion

### 3.1. Drying Kinetics

**Figure 1:**
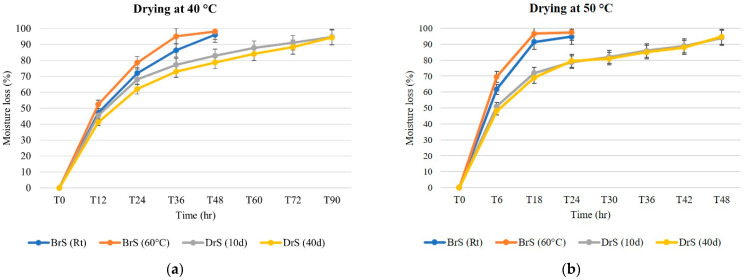
(**a**) Moisture loss % over time under 40 °C drying temperature for DrS (Rt), DrS (60 °C), BrS (10d), and BrS (40d) caper samples. (**b**) Moisture loss % over time under 50 °C drying temperatures for DrS (Rt), DrS (60 °C), BrS (10d), and BrS (40d) caper samples.

Figure 1 reports the moisture loss of the samples during the drying procedures at 40 °C and 50 °C from the different trials. The temperature significantly affected the drying rate and the total drying time, which had been reduced by half at 50 °C. Regarding the different processes, the BrS (Rt) and BrS (60 °C) samples showed similar drying kinetics and the highest drying speed, even if they had the highest initial moisture content (76.28% and 76.39%, respectively). The DrS samples had a similar behavior at both drying temperatures. Even though the DrS (10d) and DrS (40d) samples had the lowest moisture content at T0 (66.63% and 66.49%, respectively), they showed a lower drying speed than the BrS samples. This is likely due to the higher amount of added salt during the dry-salting and fermentation processes, which, due to the possible higher level of salt absorption, led to a slower degree of water release compared to the BrS samples [20]. 

### 3.2. Chemical Data

#### 3.2.1. Chlorophylls and Carotenoids

Table 1 reports the chemical compositions of the powder samples formed via different production processes and drying temperatures; the results are also compared with those from the undried capers. A significant lowest content of chlorophylls and carotenoids was achieved in the DrS (40d) samples, which were both dehydrated at 40° and 50 °C. The other samples, both DrS and BrS, showed similar content to that of the undried capers.

The levels of chlorophylls and carotenoids in our samples are in agreement with those reported in fermented capers by Tayiroğlu and İncedayı [14], who reported a higher chlorophyll content in brined capers.

During processing, heat treatment, and storage, chlorophylls and carotenoids can be easily altered or destroyed, thus modifying the perception and quality of the products [21]. In particular, chlorophylls are mainly responsible for the green color of vegetal food material; thus, their preservation is of great importance for the appearance and consumer acceptance [14,22] of vegetable powders. Moreover, it has been demonstrated that a diet rich in plant pigments, such as chlorophylls and carotenoids, plays an important role in human health [23].

#### 3.2.2. Polyphenols, Flavonoids, and Antioxidant Capacity

The polyphenol and flavonoid content and antioxidant capacity in the dried powder and undried caper samples are also reported in Table 1. In our samples, the total amounts of flavonoids and polyphenols are similar to those reported by Tayiroğlu and İncedayı [14] and Stefanucci et al. [24] for dry-salted capers. Regarding the total polyphenol content, no statistically significant differences were observed among the samples, except for the DrS samples (40d), which showed the lowest amount at both drying temperatures. The flavonoid content increased in the BrS (Rt) and BrS (60 °C) powders compared with the undried capers; for both trials, a higher temperature determined a greater flavonoid content, which was probably due to the thermal destruction of cell walls and subcellular compartments during the heat process that favored their release [25]. This behavior was opposite to that observed by Aksay et al. [20], who found a lower content in brined and dry salted samples; however, the same authors affirmed that the polyphenol and flavonoid amounts decreased following the fermentation process. In fact, as reported in literature, their amount could be influenced by several factors such as the extraction and analysis methods, harvest regions, and processing since a portion of the polyphenols and flavonoids could be released into the brine or salt [14,26,27,28,29]. 

Regarding the antioxidant capacity (AC), the DrS (10d) samples showed the highest values, even higher than the undried capers. Generally, a high drying temperature negatively influences antioxidant activity due to the degradation of thermolabile non-phenolic antioxidants [25]. In our case, the high drying temperature did not negatively influence the AC, except for the BrS (60 °C) samples.

#### 3.2.3. Aroma Compounds

Among the more than one hundred volatile compounds found in the powder samples, the main ones quantitatively represented are reported in Table 2. They belong to the class of aldehydes, alcohols, esters, ketones, terpenes, acids, sulfur compounds, pyrazines, and furanoic and aromatic compounds, of which many were previously reported by Romeo et al. [30] and Condurso et al. [9] in the capers of the Eolian Island. 

Statistically significant differences were found for most of the volatile compounds according to the production process and drying conditions. Moreover, if compared with the undried caper samples, a remarkable reduction in aromatic compounds such as benzyl alcohol, β-phenyl ethyl alcohol, and benzaldehyde was observed. Differently, the dried samples showed a high amount of sulfur compounds, mainly methyl isothiocyanate, dimethyl sulfide (sulfurous, onion, sweet, corn, vegetable, and cabbage), and aldehydes, such as 2- and 3-methyl butanal (ethereal and aldehydic), especially in the BrS (60 °C) samples. Methyl isothiocyanate represents one of the major key aroma compounds of fresh caper buds; it is synthesized by a rearrangement of intermediates that originates from glucoraphanin degradation [31]. Pyrazines, responsible for roasted notes, were only found in the dry-salted samples, both DrS (10d) and DrS (40d). These compounds arise from the Maillard reaction following heat treatment and their presence could be associated with the occurrence of a long fermentation process [32].

**Figure 2:**
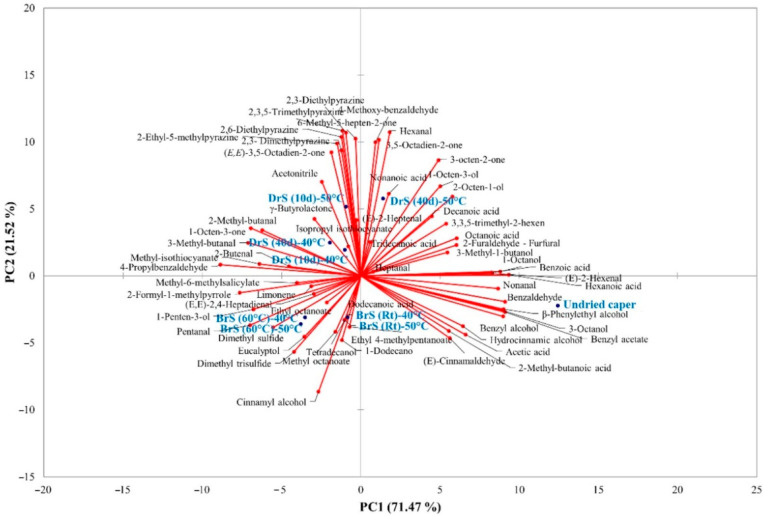
PCA loading plot showing the multivariate variation between samples in terms of volatile and aroma compounds.

To better understand the impact of the different trials and drying conditions on the volatile aroma compounds, PCA (Figure 2) was carried out. From the figure, a clear separation of the samples according to the production process resulted, with PC1 and PC2 accounting for 92.99% of the total variability. The undried capers were well-separated from the others, being alone on the positive side of PC1 and the negative side of PC2. Their separation was mainly influenced by the aromatic volatile compounds such as β-phenyl ethyl alcohol, benzyl alcohol, benzyl acetate, hydrocinnamic alcohol, and benzaldehyde, the main volatile compounds of traditional capers [9,30]. 

The dried brine samples, both BrS (Rt) and BrS (60 °C), were on the negative side of PC1 and PC2; sulfur compounds such as dimethyl sulfide and trisulfide as well as methyl isothiocyanate mostly influenced their separation. Moreover, the samples dried at different temperatures resulted in being very close one to each other, highlighting that the drying temperature lightly influenced their aroma profile.

Regarding the dry-salted samples, the DrS (10d) samples were situated on the negative side of PC1 and the positive side of PC2 together with the DrS (40d) samples dried at 40 °C: pyrazines, 2- and 3-methyl butanal, and hexanal influenced their separation. Finally, the DrS (40d) samples dried at 50 °C resulted in being on the positive side of PC1 and PC2 but very close to the DrS (10d) dried at 50 °C samples, with pyrazine, furfural, 4-methoxy benzaldehyde, acids, and ketones responsible for their separation; differently from the brined samples, the drying temperature influenced their aroma volatile profile.

#### 3.2.4. FTIR

**Figure 3:**
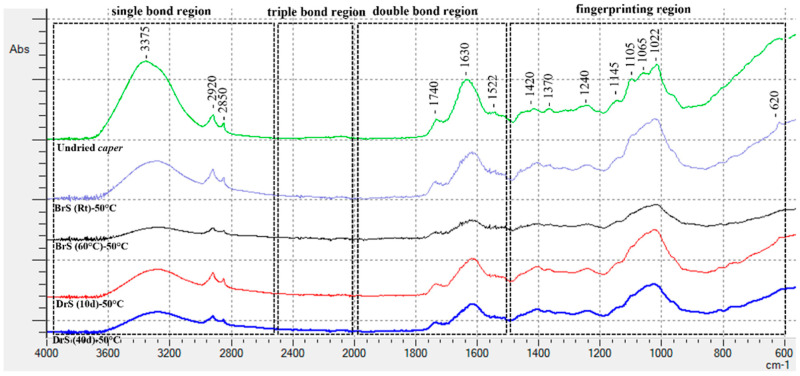
ATR-FTIR spectra of caper powders obtained from the different trials.

ATR-FTIR analysis was used to obtain qualitative information regarding the functional groups present in the samples and to find differences among them. The ATR-FTIR spectra are shown in Figure 3. The spectra of the different samples were similar in their band patterns but differed in their absorption intensity. Following the interpretation method reported by Nandiyanto et al. [33], the spectrum was divided into four regions: a single bond region (2500–4000 cm^−1^), a triple bond region (2000–2500 cm^−1^), a double bond region (1500–2000 cm^−1^), and a fingerprint region (600–1500 cm^−1^).

The broad absorption band, 3650 and 3250 cm^−1^, with a maximum absorption peak at 3375 cm^−1^, indicated a hydrogen bond related to the presence of H_2_O [34]; expectedly, the undried caper samples showed the highest absorption peak.

The narrow bands around 2920 cm^−1^ and 2850 cm^−1^ are related to the stretching C-H bond associated with the CH_2_ of lipids [35]. The lower absorption at these wavelengths observed in the BrS (60 °C) and DrS (40d) samples is likely due to the long chain fatty acid breakdown from the high brine temperature and the action of endogenous and/or microbial β-oxidation enzymes during the maturation process, respectively.

The band 1630 cm^−1^, which showed a great level of absorption in the undried DrS (40d) sample, could also be attributable to the stretching of the aromatic ring of the volatile aromatic compounds such as benzyl alcohol, β-phenyl ethyl alcohol, and benzaldehyde found in higher concentrations in these samples.

The band at 1522 cm^−1^, assigned to the stretching of C=N, C=C, and C=N of proteins, did not show variation among the samples [36].

The fingerprint region (600–1500 cm^−1^) displayed the lowest absorption intensity in the BrS (60 °C) samples. The group frequency ranging from 1000 cm^−1^ to 1300 cm^−1^ is associated with the stretching and vibration of C-O, C-OH, and C-O of many organic compounds such as esters, ethers, alcohols, and carboxylic acids; polysaccharides; cellulose; amino acids; etc. [37]. Notably, the band at 620 cm^−1^ was attributable to the stretch of the S-S bond of disulfides, which showed a higher absorption in the BrS (60 °C) sample that had the highest concentration of sulfur compounds, as reported in Table 2.

### 3.3. Sensory Analysis

#### 3.3.1. Descriptive Sensory Data

**Figure 4:**
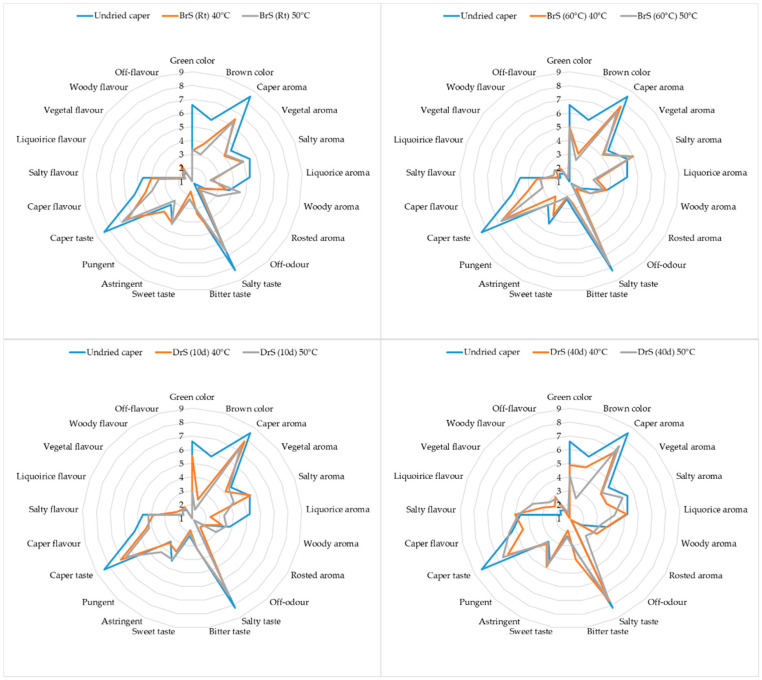
QDA sensory plot of caper powders obtained from the different trials in comparison with undried capers.

Figure 4 reports the graphical representation of the QDA sensory analysis. When compared with the undried capers, statistically significant differences can be observed regarding the green and brown colors and licorice aroma in the different trials. Among the different trials, the DrS (40d) samples, both dried at 40 and 50 °C, shoved the highest scores for brown color, licorice and roasted aroma, and licorice and roasted flavor descriptors. 

Regarding the effects of the drying temperature, differences were observed in the BrS (Rt) samples regarding a woody and roasted aroma, which was present in a lower frequency in the samples dried at 40 °C, while the samples dried at 50 °C had a more pungent taste. This could be due to the higher content of flavonoid compounds found in these samples. In the BrS (60 °C) samples dried at 50 °C, a less astringent taste and caper flavor were observed compared to those dried at 40 °C and the undried capers.

With regard to the DrS samples, more remarkable changes in sensory features were observed as an effect of the drying temperatures. The DrS (10d) samples dried at 40 °C showed had a greater green color and salty aroma than those dried at 50 °C, whereas the latter had a more licorice and roasted aroma as well as an astringent and pungent taste. This behavior could be due to the presence of pyrazines, which are responsible for roasted and licorice aromas [32]. Finally, the effect of the drying temperatures was also observed in the DrS (40d) samples regarding green and brown colors and a licorice aroma, which were higher in the samples dried at 40 °C; a salty flavor, roasted aroma, an off-odor, and caper flavor were enhanced in the capers powder dried at 50 °C.

By comparing the different trials, the main differences in the samples dried at 40 °C corresponded to a brown color, licorice aroma, caper taste, and salty flavor, wherein these levels were higher in the DrS (40d) samples, whereas these samples showed the lowest intensity regarding a salty aroma. In the samples dried at 50 °C, more intense salty, licorice, woody, vegetal, and off-flavors were observed in the DrS (40d) samples, too.

#### 3.3.2. Sensory Acceptability

Table 3 reports the results of the sensory acceptability test for the dried powders in comparison with the undried capers. Consumers expressed a greater overall acceptability for the BrS (60 °C) and DrS (40d) samples, which was comparable to that expressed for the undried capers. In contrast, the BrS (Rt) samples attained the lowest acceptance score.

From the analytical results, the powders obtained using the brine at 60 °C for three hours and then dried at 40 °C and 50 °C were characterized by a more intense vegetal and caper odor and a decreased presence of a licorice aroma, which is related to their having the highest sulfur and lowest pyrazine compound content, respectively, and by a higher amount of chlorophylls, which are mainly responsible for the green coloration. 

The different trials determined changes in the key sensory attributes, mainly color, flavor, and aroma, in relation to the type of process employed, intensity, and the raw materials used [38], thereby affecting the consumer experience [39]. Since sensory attributes and consumer acceptability are the parameters on which food industries have to base their production strategies, the BrS (60 °C) process and drying at 50 °C could represent the preferred choice for industrial caper powder production. These conditions enable the acquirement of a powder similar to undried capers and that could be preferred in terms of saving time, energy, and costs.

## 4. Conclusions

This research has developed an innovative time-, energy-, and cost-saving production process to obtain dried caper powder appreciated by consumers with interesting nutritional and sensory qualities. The most suitable process included only 6 h in brine at 60 °C and a drying period of 24 h at 50 °C, thus avoiding long fermentation and dehydration times; this process did not influence the typical aroma and sensory properties of the capers and positively increased the amount of sulfur and antioxidant compounds.

The results demonstrated that it is possible to industrially produce dried caper powder starting from fresh caper buds by avoiding the use of traditional capers, whose use is not economically advantageous. As reported herein, the fermentation process lasts forty days, thereby constituting a long period that is added to the cost and time of the drying process. In the context of the continuous growth of the dried vegetable market, the process developed herein could be of great significance to industrial production, leading to positive impacts on the economy of production areas.

## Figures and Tables

**Table 1:** Chemical data and antioxidant capacity (AC) of caper powder samples obtained from the different trials.

	BrS (Rt) 40 °C	BrS (Rt) 50 °C	40 °C vs. 50 °C	BrS (60 °C) 40 °C	BrS (60 °C) 50 °C	40 °C vs. 50 °C	DrS (10d) 40 °C	DrS (10d) 50 °C	40 °C vs. 50 °C	DrS (40d) 40 °C	DrS (40d) 50 °C	40 °C vs. 50 °C	Undried Caper
Chlorophyll a ^e^	28.21 ± 1.25 ^a^	22.15 ± 3.56 ^A^	*	22.53 ± 1.06 ^a^	18.62 ± 2.39 ^B^	*	22.27 ± 3.43 ^a^	22.36 ± 1.4 ^A^	ns	11.49 ± 0.67 ^b^	11.80 ± 0.92 ^C^	ns	25.85 ± 1.98 ^a,A^
Chlorophyll b ^e^	3.98 ± 0.73	3.21 ± 0.54 ^B^	ns	4.36 ± 0.56	4.56 ± 0.12 ^A^	ns	4.56 ± 0.29	4.67 ± 0.36 ^A^	ns	4.45 ± 0.12	2.33 ± 0.34 ^C^	*	4.01 ± 0.45 ^A^
Chlorophylls ^e^	32.19 ± 1.83 ^a^	25.36 ± 0.97 ^A^	*	26.90 ± 3.23 ^a^	23.17 ± 1.72 ^A^	ns	26.83 ± 3.85 ^a^	27.03 ± 1.72 ^A^	ns	15.94 ± 0.87 ^b^	14.13 ± 0.75 ^B^	ns	29.86 ± 1.69 ^a,A^
Carotenoids ^e^	9.44 ± 0.26 ^a^	5.80 ± 0.42 ^B^	*	4.11 ± 0.18 ^b^	9.78 ± 0.78 ^A^	**	4.97 ± 0.53 ^b^	4.99 ± 0.18 ^B^	ns	1.08 ± 0.02 ^c^	1.38 ± 0.16 ^C^	ns	8.75 ± 0.51 ^a,A^
Polyphenols ^f^	3.42 ± 0.06 ^a^	3.29 ± 0.48 ^A^	ns	3.40 ± 0.27 ^a^	3.54 ± 0.12 ^A^	ns	3.14 ± 0.23 ^a^	3.06 ± 0.21 ^A^	ns	2.77 ± 0.23 ^b^	2.39 ± 0.17 ^B^	ns	3.49 ± 0.47 ^a,A^
Flavonoids ^g^	87.00 ± 1.95 ^b^	149.21 ± 4.91 ^A^	**	99.98 ± 6.85 ^a^	117.42 ± 8.84 ^B^	*	78.08 ± 2.04 ^c^	73.43 ± 4.86 ^C^	ns	69.36 ± 3.98 ^d^	53.66 ± 1.03 ^C^	ns	71.24 ± 3.24 ^c,C^
AC (%) ^h^	80.66 ± 2.35 ^b^	77.33 ± 3.38 ^B^	ns	83.33 ± 4.58 ^a^	75.55 ± 4.92 ^B^	*	86.88 ± 3.94 ^a^	86.66 ± 2.12 ^A^	ns	81.55 ± 2.37 ^b^	80.88 ± 2.76 ^B^	ns	81.77 ± 5.18 ^b,B^

ANOVA: ** represents significant differences at *p* < 0.01, * represents significant differences at *p* < 0.05, and ns = not statistically significant (*p* > 0.5) between samples obtained by the same trial dried at 40 and 50 °C; ^a–c^ different superscript lower case letters in the same row represent significant differences at *p* < 0.05 determined by Duncan’s multiple range test among samples of different trials dried at 40 °C and undried capers; ^A–C^ different superscript capital letters in the same row represent significant differences at *p* < 0.05 determined by Duncan’s multiple range test among samples of different trials dried at 50 °C and undried capers; ^e^ mg/100g dry matter; ^f^ mgEQ gallic acid/g dry matter; ^g^ mgEQ quercetin/g dry matter; ^h^ total antioxidant capacity expressed as % of DPPH inhibition.

**Table 2:** Volatile aroma compounds (Area %) identified in caper powder samples obtained from the different trials.

	LRI	BrS (Rt) 40 °C	BrS (Rt) 50 °C	40 °C vs. 50 °C	BrS (60 °C) 40 °C	BrS (60 °C) 50 °C	40 °C vs. 50 °C	DrS (10d) 40 °C	DrS (10d) 50 °C	40 °C vs. 50 °C	DrS (40d) 40 °C	DrS (40d) 50 °C	40 °C vs. 50 °C	Undried Caper
Aldehydes														
2-Methyl-butanal	917	3.65 ± 0.15 ^b^	5.44 ± 0.27 ^A^	*	2.55 ± 0.11 ^b^	3.84 ± 0.26 ^B^	*	2.78 ± 0.17 ^b^	3.77 ± 0.24 ^B^	*	6.04 ± 0.37 ^a^	4.70 ± 0.31 ^A^	*	0.10 ± 0.02 ^c,C^
3-Methyl-butanal	920	4.21 ± 0.25 ^b^	6.30 ± 0.28 ^A^	*	5.26 ± 0.46 ^b^	7.87 ± 0.81 ^A^	*	4.48 ± 0.29 ^b^	4.95 ± 0.31 ^B^	ns	9.92 ± 0.73 ^a^	6.52 ± 0.43 ^A^	*	0.39 ± 0.02 ^c,C^
Pentanal	983	1.59 ± 0.08 ^b^	2.22 ± 0.21 ^B^	*	4.16 ± 0.29 ^a^	3.91 ± 0.26 ^A^	ns	2.04 ± 0.09 ^b^	1.73 ± 0.09 ^B^	*	2.76 ± 0.19 ^b^	1.85 ± 0.84 ^B^	*	0.86 ± 0.08 ^c,C^
2-Butenal	1044	0.41 ± 0.01 ^b^	0.34 ± 0.02 ^A^	ns	0.84 ± 0.05 ^a^	0.40 ± 0.03 ^A^	*	0.40 ± 0.02 ^b^	0.35 ± 0.02 ^A^	ns	0.89 ± 0.06 ^a^	0.46 ± 0.05 ^A^	*	0.26 ± 0.03 ^c,B^
Hexanal	1081	1.43 ± 0.09 ^b^	2.12 ± 0.16 ^C^	*	1.86 ± 0.09 ^b^	1.19 ± 0.13 ^C^	*	3.04 ± 0.27 ^a^	5.12 ± 0.36 ^B^	*	3.96 ± 0.27 ^a^	7.12 ± 0.40 ^A^	*	2.90 ± 0.21 ^a,C^
Heptanal	1185	0.56 ± 0.03	0.58 ± 0.04 ^A^	ns	0.39 ± 0.02	0.21 ± 0.03 ^B^	*	0.42 ± 0.02	0.57 ± 0.03 ^A^	ns	0.35 ± 0.02	0.34 ± 0.02 ^B^	ns	0.41 ± 0.02 ^B^
(E)-2-Hexenal	1221	0.22 ± 0.01 ^c^	0.24 ± 0.02 ^C^	ns	0.44 ± 0.02 ^b^	0.31 ± 0.02 ^C^	ns	0.23 ± 0.01 ^c^	0.34 ± 0.02 ^C^	ns	0.41 ± 0.03 ^b^	0.53 ± 0.03 ^B^	ns	0.97 ± 0.04 ^a,A^
(E)-2-Heptenal	1326	1.03 ± 0.12	0.99 ± 0.06	ns	1.62 ± 0.11	1.05 ± 0.13	*	0.79 ± 0.08	1.36 ± 0.07	*	1.35 ± 0.18	1.58 ± 0.12	ns	1.18 ± 0.09
Nonanal	1393	0.57 ± 0.34 ^b^	0.72 ± 0.04 ^B^	*	0.59 ± 0.03 ^b^	0.72 ± 0.05 ^B^	ns	0.73 ± 0.03 ^b^	0.88 ± 0.06 ^B^	ns	0.70 ± 0.04 ^b^	0.65 ± 0.08 ^B^	ns	1.58 ± 0.13 ^a,A^
(E,E)-2,4-Heptadienal	1494	0.31 ± 0.26 ^b^	0.28 ± 0.02 ^B^	ns	0.74 ± 0.05 ^a^	0.67 ± 0.04 ^A^	ns	0.34 ± 0.02 ^b^	0.34 ± 0.02 ^B^	ns	0.55 ± 0.04 ^b^	0.54 ± 0.03 ^A^	ns	0.41 ± 0.05 ^b,B^
All		13.99	19.23		18.44	20.16		15.24	19.41		26.93	24.29		9.04
Alcohols														
1-Penten-3-ol	1157	0.64 ± 0.04 ^b^	0.71 ± 0.04 ^A^	ns	0.82 ± 0.03 ^a^	0.90 ± 0.06 ^A^	ns	0.30 ± 0.02 ^c^	0.63 ± 0.04 ^A^	**	0.50 ± 0.03 ^b^	0.69 ± 0.07 ^A^	ns	0.21 ± 0.01 ^c,B^
3-Methyl-1-butanol	1204	0.13 ± 0.02 ^c^	0.29 ± 0.02 ^A^	*	0.16 ± 0.02 ^c^	- ^B^	*	0.23 ± 0.03 ^b^	0.27 ± 0.02 ^A^	ns	0.21 ± 0.02 ^b^	0.16 ± 0.02 ^A^	ns	0.31 ± 0.02 ^a,A^
3-Octanol	1388	- ^b^	- ^B^	ns	- ^b^	- ^B^	ns	- ^b^	- ^B^	ns	- ^b^	- ^B^	ns	0.30 ± 0.02 ^a,A^
1-Octen-3-ol	1444	1.15 ± 0.16 ^b^	1.07 ± 0.23 ^C^	ns	0.72 ± 0.04 ^c^	0.47 ± 0.03 ^D^	*	0.55 ± 0.03 ^c^	2.03 ± 0.14 ^B^	**	0.84 ± 0.06 ^b^	3.92 ± 0.21 ^A^	**	2.30 ± 0.17 ^a,B^
1-Octanol	1551	0.18 ± 0.02 ^a^	0.26 ± 0.02 ^A^	*	0.26 ± 0.02 ^a^	0.16 ± 0.02 ^A^	*	0.21 ± 0.01 ^a^	0.23 ± 0.04 ^A^	ns	0.25 ± 0.03 ^a^	0.31 ± 0.02 ^A^	ns	0.50 ± 0.06 ^a,A^
2-Octen-1-ol	1610	0.13 ± 0.01 ^C^	0.14 ± 0.01 ^C^	ns	0.19 ± 0.01 ^c^	0.10 ± 0.02 ^C^	*	0.11 ± 0.01 ^C^	0.31 ± 0.02 ^B^	*	0.22 ± 0.01 ^B^	0.93 ± 0.08 ^A^	*	0.62 ± 0.04 ^A,a^
Tetradecanol	2173	0.20 ± 0.02	0.20 ± 0.01	ns	0.17 ± 0.02	0.17 ± 0.03	ns	0.14 ± 0.02	0.23 ± 0.03	*	0.18 ± 0.02	0.16 ± 0.01	ns	0.15 ± 0.03
All		2.44	2.66		2.32	1.80		1.54	3.69		2.21	6.16		4.38
Esters														
Ethyl 4-methylpentanoate	1185	0.56 ± 0.04 ^a^	0.04 ± 0.01 ^A^	**	- ^b^	- ^B^	ns	- ^b^	- ^B^	ns	- ^b^	- ^B^	ns	- ^b,B^
Methyl octanoate	1388	1.71 ± 0.23 ^a^	1.79 ± 0.23 ^A^	ns	0.04 ± 0.01 ^b^	0.10 ± 0.01 ^C^	*	0.13 ± 0.02 ^b^	0.51 ± 0.04 ^B^	**	0.11 ± 0.02 ^b^	0.08 ± 0.01 ^C^	ns	- ^c,D^
Ethyl octanoate	1435	0.47 ± 0.03 ^a^	0.53 ± 0.06 ^A^	ns	- ^c^	- ^C^	ns	0.18 ± 0.03 ^b^	0.25 ± 0.03 ^B^	ns	0.19 ± 0.03 ^b^	- ^C^	**	- ^c,C^
All		2.74	2.36		0.04	0.10		0.31	0.76		0.30	0.08		-
Ketones														
1-Octen-3-one	1303	0.23 ± 0.02 ^a^	0.21 ± 0.02 ^A^	ns	0.33 ± 0.02 ^a^	0.26 ± 0.03 ^A^	ns	0.18 ± 0.02 ^a^	0.32 ± 0.03 ^A^	*	0.28 ± 0.01 ^a^	0.28 ± 0.02 ^A^	ns	0.11 ± 0.01 ^b,B^
6-Methyl-5-hepten-2-one	1337	0.60 ± 0.03	0.60 ± 0.03	ns	0.64 ± 0.03	0.70 ± 0.04	ns	0.72 ± 0.04	0.80 ± 0.04	ns	0.81 ± 0.04	0.81 ± 0.03	ns	0.67 ± 0.04
3-octen-2-one	1409	0.08 ± 0.01 ^c^	- ^C^	*	0.03 ± 0.01 ^c^	0.07 ± 0.01 ^B^	ns	0.15 ± 0.02 ^b^	0.27 ± 0.02 ^A^	*	0.20 ± 0.01 ^b^	0.57 ± 0.03 ^A^	*	0.33 ± 0.02 ^a,A^
(E,Z)-3,5-Octadien-2-one	1521	0.39 ± 0.02 ^b^	0.40 ± 0.03 ^C^	ns	0.68 ± 0.04 ^a^	1.03 ± 0.10 ^B^	*	0.76 ± 0.06 ^a^	1.61 ± 0.12 ^A^	*	1.00 ± 0.05 ^a^	1.29 ± 0.09 ^A^	ns	0.55 ± 0.03 ^b,C^
(E,E)-3,5-Octadien-2-one	1569	1.14 ± 0.15 ^b^	0.49 ± 0.03 ^C^	**	1.13 ± 0.17 ^b^	1.36 ± 0.21 ^B^	ns	1.35 ± 0.16 ^b^	2.91 ± 0.19 ^A^	*	2.65 ± 0.13 ^a^	3.00 ± 0.11 ^A^	ns	1.69 ± 0.07 ^b,B^
All		2.43	1.70		2.81	3.42		3.16	5.91		4.93	5.95		3.35
Terpenes														
Limonene	1197	0.22 ± 0.01 ^c^	0.63 ± 0.03 ^A^	*	0.85 ± 0.03 ^a^	0.21 ± 0.01 ^B^	**	0.87 ± 0.04 ^a^	0.30 ± 0.02 ^B^	*	0.54 ± 0.02 ^b^	0.48 ± 0.03 ^A^	ns	0.24 ± 0.03 ^c,B^
Eucalyptol	1208	0.04 ± 0.01 ^b^	0.13 ± 0.01	*	0.33 ± 0.04 ^a^	0.07 ± 0.03	*	0.12 ± 0.01 ^b^	0.01 ± 0.01	**	0.08 ± 0.01 ^b^	0.06 ± 0.01	ns	0.03 ± 0.01 ^b^
All		0.26	0.77		1.18	0.27		0.75	0.31		0.62	0.55		0.28
Acids														
Acetic acid	1453	1.38 ± 0.11 ^b^	2.43 ± 0.15 ^A^	*	0.42 ± 0.03 ^d^	0.26 ± 0.01 ^C^	*	0.74 ± 0.05 ^c^	0.54 ± 0.03 ^B^	*	0.62 ± 0.04 ^c^	0.80 ± 0.06 ^B^	ns	2.51 ± 0.14 ^a,A^
2-Methyl-butanoic acid	1668	0.86 ± 0.04 ^a^	0.81 ± 0.03 ^A^	ns	0.16 ± 0.02 ^b^	0.23 ± 0.01 ^C^	*	0.24 ± 0.02 ^b^	0.21 ± 0.01 ^C^	ns	0.37 ± 0.02 ^b^	0.51 ± 0.04 ^B^	ns	0.84 ± 0.02 ^a,A^
Hexanoic acid	1851	0.75 ± 0.06 ^b^	0.49 ± 0.02 ^C^	*	0.12 ± 0.01 ^c^	0.15 ± 0.01 ^D^	ns	0.54 ± 0.03 ^b^	0.38 ± 0.02 ^C^	ns	0.80 ± 0.05 ^b^	1.16 ± 0.14 ^B^	*	2.90 ± 0.16 ^a,A^
Octanoic acid	2064	6.82 ± 0.23 ^a^	7.37 ± 0.26 ^A^	ns	1.13 ± 0.10 ^b^	1.10 ± 0.14 ^C^	ns	6.27 ± 0.27 ^a^	5.57 ± 0.24 ^B^	ns	7.59 ± 0.38 ^a^	5.91 ± 0.32 ^B^	*	8.93 ± 0.41 ^a,A^
Nonanoic acid	2169	0.39 ± 0.02	0.40 ± 0.03 ^B^	ns	0.30 ± 0.02	0.29 ± 0.03 ^B^	ns	0.26 ± 0.02	0.36 ± 0.04 ^B^	ns	0.37 ± 0.02	0.84 ± 0.05 ^A^	*	0.36 ± 0.02 ^B^
Decanoic acid	2275	0.28 ± 0.01 ^a^	0.12 ± 0.01 ^C^	*	0.10 ± 0.01 ^b^	0.09 ± 0.01 ^C^	ns	0.10 ± 0.01 ^b^	0.19 ± 0.01 ^B^	*	0.13 ± 0.01 ^b^	0.34 ± 0.02 ^A^	*	0.23 ± 0.01 ^a,B^
Dodecanoic acid	2427	0.88 ± 0.04 ^a^	0.24 ± 0.02 ^B^	*	0.17 ± 0.02 ^b^	0.13 ± 0.02 ^C^	ns	0.12 ± 0.01 ^b^	0.19 ± 0.02 ^C^	*	0.11 ± 0.01 ^b^	0.43 ± 0.03 ^A^	**	0.14 ± 0.01 ^b,C^
Tridecanoic acid	2553	0.69 ± 0.05 ^a^	0.34 ± 0.02 ^B^	*	0.98 ± 0.05 ^a^	0.16 ± 0.01 ^C^	*	0.32 ± 0.02 ^b^	0.40 ± 0.03 ^B^	ns	0.19 ± 0.02 ^b^	1.26 ± 0.08 ^A^	**	0.44 ± 0.03 ^b,B^
All		12.05	12.21		3.37	2.42		8.59	7.84		10.17	11.24		16.36
Sulfur compounds														
Dimethyl sulfide	745	1.08 ± 0.07 ^b^	2.71 ± 0.18 ^B^	*	10.85 ± 1.97 ^a^	16.01 ± 1.02 ^A^	*	2.48 ± 0.12 ^b^	1.41 ± 0.09 ^B^	*	9.19 ± 0.71 ^a^	2.07 ± 0.15 ^B^	**	0.18 ± 0.01 ^c,C^
Isopropyl isothiocyanate	1186	- ^b^	-		- ^b^	-	ns	0.42 ± 0.02 ^a^	-	**	- ^b^	-	ns	- ^b^
Methyl-isothiocyanate	1241	12.81 ± 0.87 ^b^	15.47 ± 1.17 ^B^	ns	28.01 ± 1.39 ^a^	24.89 ± 1.23 ^A^	ns	33.30 ± 1.86 ^a^	28.49 ± 2.26 ^A^	*	12.91 ± 0.95 ^b^	9.35 ± 0.74 ^B^	*	3.06 ± 0.17 ^c,C^
Dimethyl trisulfide	1386	- ^b^	- ^B^		0.03 ± 0.01 ^a^	0.04 ± 0.01 ^A^	ns	- ^b^	- ^B^	ns	- ^b^	- ^B^		- ^b,B^
All		13.90	18.18		38.89	40.95		36.19	29.90		22.10	11.41		3.24
Pyrazines														
2,3- Dimethylpyrazine	1348	- ^b^	- ^B^	ns	- ^b^	- ^B^	ns	0.53 ± 0.03 ^a^	0.26 ± 0.02 ^A^	*	0.32 ± 0.02 ^a^	0.32 ± 0.04 ^A^	ns	- ^b,B^
2-Ethyl-5-methylpyrazine	1387	- ^b^	- ^B^	ns	- ^b^	- ^B^	ns	0.25 ± 0.01 ^a^	0.40 ± 0.03 ^A^	*	0.20 ± 0.03 ^a^	0.25 ± 0.01 ^A^	ns	- ^b,B^
2,3,5-Trimethylpyrazine	1405	- ^b^	- ^B^	ns	- ^b^	- ^B^	ns	0.12 ± 0.02 ^a^	0.17 ± 0.02 ^A^	ns	0.09 ± 0.01 ^a^	0.11 ± 0.02 ^A^	ns	- ^b,B^
2,6-Diethylpyrazine	1433	- ^b^	- ^C^	ns	- ^b^	- ^C^	ns	0.26 ± 0.03 ^a^	0.43 ± 0.03 ^A^	*	0.19 ± 0.02 ^a^	0.20 ± 0.03 ^B^	ns	- ^b,C^
2,3-Diethylpyrazine	1459	- ^b^	- ^B^	ns	- ^b^	- ^B^	ns	0.12 ± 0.01 ^a^	0.19 ± 0.02 ^A^	ns	0.07 ± 0.01 ^a^	0.12 ± 0.01 ^A^	ns	- ^b,B^
All		-	-		-	-		1.29	1.46		0.86	1.00		-
Furanoic compounds														
Furfural	1466	0.28 ± 0.03 ^b^	0.22 ± 0.01 ^B^	ns	0.39 ± 0.05 ^a^	0.34 ± 0.02 ^B^	ns	0.19 ± 0.01 ^b^	0.28 ± 0.02 ^B^	ns	0.49 ± 0.05 ^a^	0.59 ± 0.06 ^A^	ns	0.64 ± 0.05 ^a,A^
γ-Butyrolactone	1632	0.33 ± 0.02 ^b^	1.98 ± 0.14 ^A^	**	0.09 ± 0.01 ^c^	0.08 ± 0.01 ^C^	ns	0.73 ± 0.06 ^a^	0.40 ± 0.03 ^B^	*	0.23 ± 0.03 ^b^	0.22 ± 0.01 ^C^	ns	- ^d,D^
All		0.61	2.20		0.47	0.41		0.91	0.68		0.72	0.81		0.64
Aromatic compounds														
Aldehydes														
Benzaldehyde	1525	1.59 ± 0.26 ^b^	1.68 ± 0.16 ^B^	ns	1.46 ± 0.15 ^b^	1.63 ± 0.08 ^B^	ns	1.51 ± 0.10 ^b^	1.89 ± 0.09 ^B^	ns	1.40 ± 0.11 ^b^	1.72 ± 0.03 ^B^	ns	4.47 ± 0.21 ^a,A^
4-Propylbenzaldehyde	1826	0.69 ± 0.05 ^a^	1.07 ± 0.09 ^A^	*	1.11 ± 0.21 ^a^	1.15 ± 0.09 ^A^	ns	0.78 ± 0.06 ^a^	1.07 ± 0.12 ^A^	*	0.95 ± 0.05 ^a^	0.82 ± 0.05 ^A^	ns	0.10 ± 0.02 ^b,B^
4-Methoxy-benzaldehyde	2029	0.65 ± 0.03 ^c^	0.84 ± 0.05 ^B^	ns	0.50 ± 0.04 ^c^	0.65 ± 0.05 ^C^	ns	2.07 ± 0.15 ^a^	1.69 ± 0.04 ^A^	ns	1.97 ± 0.06 ^a^	1.75 ± 0.16 ^A^	ns	1.11 ± 0.10 ^b,B^
(E)-Cinnamaldehyde	2044	0.09 ± 0.05 ^c^	0.16 ± 0.02 ^C^	ns	0.25 ± 0.01 ^b^	0.37 ± 0.04 ^B^	*	0.26 ± 0.03 ^b^	0.16 ± 0.01 ^C^	ns	0.17 ± 0.01 ^b^	0.10 ± 0.01 ^C^	ns	0.54 ± 0.04 ^a,A^
Alcohols														
Benzyl alcohol	1877	1.91 ± 0.15 ^b^	2.10 ± 0.16 ^B^	ns	1.14 ± 0.23 ^b^	1.44 ± 0.10 ^B^	ns	1.63 ± 0.09 ^b^	0.91 ± 0.04 ^C^	*	1.31 ± 0.15 ^b^	1.10 ± 0.06 ^C^	ns	20.13 ± 1.21 ^a,A^
β-Phenylethyl alcohol	1912	0.08 ± 0.01 ^b^	0.08 ± 0.01 ^B^	ns	0.05 ± 0.01 ^b^	0.07 ± 0.01 ^B^	ns	0.13 ± 0.01 ^b^	0.07 ± 0.02 ^B^	ns	0.13 ± 0.02 ^b^	0.12 ± 0.02 ^B^	ns	6.59 ± 0.43 ^a,A^
Hydrocinnamic alcohol	2047	- ^c^	0.67 ± 0.05 ^A^	*	0.27 ± 0.02 ^b^	0.28 ± 0.03 ^B^	ns	0.31 ± 0.03 ^b^	0.27 ± 0.03 ^B^	ns	0.29 ± 0.03 ^b^	0.11 ± 0.01 ^C^	ns	0.91 ± 0.04 ^a,A^
Cinnamyl alcohol	2287	0.44 ± 0.02 ^b^	1.30 ± 0.16 ^A^	*	1.15 ± 0.14 ^a^	1.86 ± 0.15 ^A^	*	0.37 ± 0.02 ^c^	0.28 ± 0.03 ^C^	ns	0.24 ± 0.02 ^c^	0.22 ± 0.03 ^C^	ns	0.65 ± 0.02 ^b,B^
Ketones														
Acetophenone	1663	0.42 ± 0.04 ^a^	0.70 ± 0.04 ^A^	*	0.25 ± 0.01 ^b^	0.21 ± 0.03 ^B^	ns	0.12 ± 0.02 ^b^	0.15 ± 0.02 ^B^	ns	0.21 ± 0.01 ^b^	0.32 ± 0.05 ^B^	ns	0.13 ± 0.01 ^b,B^
Esters														
Benzyl acetate	1728	0.10 ± 0.01 ^b^	0.14 ± 0.02 ^B^	ns	0.03 ± 0.01 ^b^	0.04 ± 0.01 ^C^	ns	0.08 ± 0.01 ^b^	0.08 ± 0.01 ^C^	ns	0.08 ± 0.01 ^b^	0.06 ± 0.01 ^C^	ns	0.72 ± 0.04 ^a,A^
Methyl-6-methylsalicylate	1965	0.41 ± 0.05 ^a^	0.81 ± 0.06 ^A^	*	0.29 ± 0.03 ^b^	0.38 ± 0.04 ^B^	*	0.47 ± 0.05 ^a^	0.43 ± 0.03 ^B^	ns	0.43 ± 0.05 ^a^	0.38 ± 0.05 ^B^	ns	0.19 ± 0.02 ^c,C^
Acids														
Benzoic acid	2401	- ^c^	- ^D^	ns	- ^c^	0.26 ± 0.03 ^B^	**	0.37 ± 0.02 ^b^	0.06 ± 0.01 ^C^	**	- ^c^	0.40 ± 0.06 ^B^	*	0.95 ± 0.06 ^a,A^
All		6.38	9.53		6.50	8.34		8.12	7.07		7.18	7.09		36.49
Nitrogen compounds														
Acetonitrile	1010	0.36 ± 0.04 ^a^	- ^C^	*	0.02 ± 0.01 ^b^	0.03 ± 0.01 ^B^	ns	0.37 ± 0.03 ^a^	0.52 ± 0.03 ^A^	ns	0.42 ± 0.03 ^a^	0.13 ± 0.02 ^B^	*	- ^c,C^
2-Formyl-1-methyl pyrrole	1621	0.54 ± 0.03 ^a^	0.83 ± 0.04 ^A^	*	0.62 ± 0.04 ^a^	0.99 ± 0.05 ^A^	*	0.45 ± 0.03 ^a^	0.41 ± 0.02 ^B^	ns	0.74 ± 0.05 ^a^	0.71 ± 0.06 ^A^	ns	- ^b,C^
All		0.90	0.83		0.64	1.03		0.83	0.93		1.16	0.84		-

ANOVA: ** represents significant differences at *p* < 0.01, * represents significant differences at *p* < 0.05, and ns = not statistically significant (*p* > 0.5) between samples obtained by the same trial dried at 40 and 50 °C; - = not detected; ^a–d^ different superscript lower case letters in the same row represent significant differences at *p* < 0.05 determined by Duncan’s multiple range test among samples of different trials dried at 40 °C and undried capers; ^A–D^ different superscript capital letters in the same row represent significant differences at *p* < 0.05 determined by Duncan’s multiple range test among samples of different trials dried at 50 °C and undried capers.

**Table 3:** Sensory acceptability of caper powder samples obtained from the different trials.

	BrS (Rt) 40 °C	BrS (Rt) 50 °C	40 °C vs. 50 °C	BrS (60 °C) 40 °C	BrS (60 °C) 50 °C	40 °C vs. 50 °C	DrS (10d) 40 °C	DrS (10d) 50 °C	40 °C vs. 50 °C	DrS (40d) 40 °C	DrS (40d) 50 °C	40 °C vs. 50 °C	Undried Caper
Appearance	6.38 ± 0.12 ^b^	6.75 ± 0.07 ^C^	ns	7.49 ± 0.15 ^a^	7.20 ± 0.07 ^B^	ns	6.88 ± 0.05 ^b^	6.25 ± 0.06 ^C^	ns	7.57 ± 0.03 ^a^	6.71 ± 0.04 ^B^	*	7.80 ± 0.05 ^a,A^
Odor	4.50 ± 0.09 ^b^	4.88 ± 0.20 ^B^	ns	7.14 ± 0.06 ^a^	6.71 ± 0.13 ^A^	*	5.88 ± 0.07 ^b^	5.63 ± 0.08 ^A^	ns	6.86 ± 0.17 ^a^	6.96 ± 0.15 ^A^	ns	6.90 ± 0.16 ^a,A^
Taste	4.25 ± 0.02 ^b^	5.00 ± 0.16 ^B^	*	8.00 ± 0.23 ^a^	8.29 ± 0.25 ^A^	ns	5.38 ± 0.14 ^b^	5.25 ± 0.24 ^B^	ns	7.71 ± 0.18 ^a^	7.86 ± 0.21 ^A^	ns	7.40 ± 0.19 ^a,A^
Overall acceptability	5.04 ± 0.18 ^b^	5.54 ± 0.05 ^B^	ns	7.54 ± 0.16 ^a^	7.40 ± 0.08 ^A^	ns	6.04 ± 0.18 ^b^	5.71 ± 0.07 ^B^	ns	7.38 ± 0.13 ^a^	7.17 ± 0.09 ^A^	ns	7.37 ± 0.07 ^a,A^

ANOVA: * represents significant differences at *p* < 0.05, and ns = not statistically significant (*p* > 0.5) between samples obtained by the same trial dried at 40 and 50 °C; ^a–b^ different superscript lower case letters in the same row represent significant differences at *p* < 0.05 determined by Duncan’s multiple range test among samples of different trials dried at 40 °C and undried capers; ^A–C^ different superscript capital letters in the same row represent significant differences at *p* < 0.05 determined by Duncan’s multiple range test among samples of different trials dried at 50 °C and undried capers.

## Data Availability

The data presented in this study are available on request from the corresponding author.
